# Exploring the Extent in the Visual Field of the Honeycomb and Extinction Illusions

**DOI:** 10.1177/2041669519854784

**Published:** 2019-07-04

**Authors:** Marco Bertamini, Aline F. Cretenoud, Michael H. Herzog

**Affiliations:** Department of Psychological Sciences, University of Liverpool, UK; Laboratory of Psychophysics, Brain Mind Institute, École Polytechnique Fédérale de Lausanne (EPFL), Switzerland

**Keywords:** texture, crowding, peripheral vision, suppression, Honeycomb illusion, Extinction illusion

## Abstract

There are situations in which what is perceived in central vision is different to what is perceived in the periphery, even though the stimulus display is uniform. Here, we studied two cases, known as the Extinction illusion and the Honeycomb illusion, involving small disks and lines, respectively, presented over a large extent of the visual field. Disks and lines are visible in the periphery on their own, but they become invisible when they are presented as part of a pattern (grid). Observers (*N* = 56) adjusted a circular probe to report the size of the region in which they had seen the lines or the disks. Different images had black or white lines/disks, and we included control stimuli in which these features were spatially separated from the regular grid of squares. We confirmed that the illusion was experienced by the majority of observers and is dependent on the interaction between the elements (i.e., the lines/disks have to be near the squares). We found a dissociation between the two illusions in the dependence on contrast polarity suggesting different mechanisms. We analysed the variability between individuals with respect to schizotypical and autistic-spectrum traits (short version of the Oxford-Liverpool Inventory of Feelings and Experiences [O-LIFE] questionnaire and the Autistic Quotient, respectively) but found no significant relationships. We discuss how illusions relative to what observers are aware of in the periphery may offer a unique tool to study visual awareness.

For humans, the visual field extends approximately 180° horizontally and 160° vertically. However, only parts of the visual field are binocular, a large blind spot is present in each hemifield, and both resolution and colour vision vary greatly with eccentricity (Helmholtz, 1867). Our experience, nevertheless, is that of a uniform and stable visual field, possibly because of the properties perceived in the central region (Gibson, 1950). This experience of a detailed and uniform visual field can be described as an illusion, perhaps the most striking of all visual illusions. This has been studied by psychologists and commented on by philosophers and is sometimes called the *grand illusion hypothesis* (Blackmore, Brelstaff, Nelson, & Trościanko, 1995; Dennett, 1991; Noë, Pessoa, & Thompson, 2000; Rensink, O’Regan, & Clark, 1997).

A large literature has explored, both theoretically and empirically, to what extent summary statistical information can surmount the limitations imposed by the visual system on the representation of individual elements (Chong & Treisman, 2003; Cohen, Dennett, & Kanwisher, 2016; Dehaene, 2014; Lamme, 2010). A summary or ensemble representation implies that sets of similar items are coded only in terms of summary statistics (Haberman & Whitney, 2012). McClelland and Bayne (2016) have proposed a distinction between summary statistics that observers are aware of, although they may be lost in memory, and a second type of summary representations that are not part of phenomenal experience, although they can affect post-perceptual judgements. It is known that early visual responses are combined in receptive fields that grow with eccentricity. Freeman and Simoncelli (2011) have developed a model and created visual metamers: stimuli that differ physically in the periphery but look the same.

To study what observers are aware of in the periphery researchers have developed various procedures. There are also some visual illusions that provide useful insights.

## Honeycomb and Extinction Illusions

**Figure 1.**
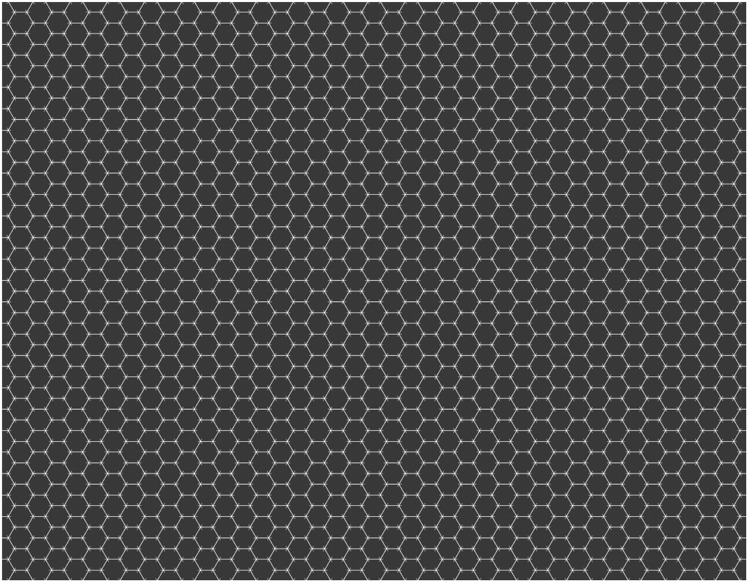
This texture has hexagons and small lines at the vertices of the hexagons. The texture is uniform, but it is not perceived as such because the lines can only be seen at or near fixation. The image needs to be enlarged or looked at closely, so as to fill a large proportion of the visual field; for more examples, go to https://osf.io/kabyz/

Recently, Bertamini, Herzog, and Bruno (2016) have described an illusion characterised by the inability of seeing shapes in the periphery, when these shapes are part of a texture, as shown in Figure 1. The uniform pattern (hexagons and lines) extends over a large part of the visual field, but in the periphery, observers are not aware of some of its features (lines). Therefore, a physically uniform stimulus yields a non-uniform percept. Bertamini et al. (2016) named this effect the Honeycomb (HC) illusion and we will use the acronym HC. In Figure 1, the small lines are visible at fixation, but invisible everywhere else. This is true even after multiple fixations, and the phenomenon is robust to changes in shape and size of the elements. Because the experience does not change over time and with multiple fixations, memory seems to have no role in the build-up and maintenance of the representation of the visual field. Moreover, this representation is not an extrapolation from information available to central vision.

A closely related phenomenon is known as the Extinction (EX) illusion (Ninio & Stevens 2000). This was described as a variation on the Hermann grid (HG), but it is in fact a striking phenomenon on its own. We will use the shorten name EX illusion. As for the HC illusion, features in the periphery completely disappear from awareness. In the case of the EX illusion, they are local disks at the intersection of lines. For a strong effect, the disks have to be light (e.g., light grey) on a mainly dark background (e.g. black) or vice versa.

The experimental evidence about these illusions is limited with a few notable exceptions. McAnany and Levine (2004) and Levine, Anderson, and McAnany (2012) reported a series of experiments on a phenomenon that they call *blanking*. This is closely related to the EX illusion in that a light disk becomes invisible when presented at an intersection of black squares forming a grid. They demonstrated that the disk and at least four squares of the grid must be presented simultaneously. Levine et al. (2012) discovered also that distorting the squares, that is, making the alleys wavy, makes the illusion stronger. Levine and McAnany (2008) have argued that there are two effects for disks at intersections of a grid with dark squares: An obscuring process affects every target in a grid, while blanking only affects the light disks. What that means is that the blanking, in agreement with Ninio and Stevens (2000) observations, is strongest when there is a difference in polarity between the patterns of squares and the disks: When the squares are black, the disks need to be white or vice versa.

Common to all these effects, and in particular to the HC and EX illusions, is that they are instantaneous and do not require any adaptation period. However, their strength may vary over time. Araragi and Kitaoka (2011) found that the EX illusion becomes stronger with longer presentations. At short presentations, they also reported an anisotropy: The illusion occurred more frequently in the upper visual field than in the lower visual field.

Note that these illusions show the opposite of an extrapolation effect. What is visible in central vision is not extrapolated, even though that would produce a veridical percept. Extrapolation effects have been reported, but they seem to require extended presentation time and adaptation (Otten, Pinto, Paffen, Seth, & Kanai, 2017). An example of an adaptation phenomenon is the Healing grid illusion in which the regularity present at central fixation becomes a property of the grid in the periphery over time (Kanai, 2005). In discrimination tasks, there is also evidence that foveal information can affect judgements of shape (Yu & Shim, 2016) and of brightness (Toscani, Gegenfurtner, & Valsecchi, 2017) in the periphery. Gloriani and Schütz (2019) have reported evidence that observers trust what is perceived in central vision both in photopic and, surprisingly, in scotopic vision.

One of the best known and most studied effects about vision in the periphery is the HG illusion (Geier, Bernáth, Hudák, & Séra, 2008; Hermann, 1870). The full explanation of the HG phenomenon is still under study and is more complex than a process of lateral inhibition (Qian, Yamada, Kawabe, & Miura, 2009; Read, Robson, Smith, & Lucas, 2012; Schiller & Carvey, 2005). Some variants of the HG are especially interesting and constitute distinct phenomena. In particular, blurring the HG changes the dark smudges into scintillating black spots (Bergen, 1985; Schrauf, Lingelbach, Lingelbach, & Wist, 1995; Schrauf, Lingelbach, & Wist, 1997). Other studies have reported shape distortions: Disks become ellipses away from fixation (Qian & Mitsudo, 2016).

Like the HC and the EX illusions, the HG effect is instantaneous and requires an extended grid. It is also similar in that what is seen in central vision is different from what is experienced in the periphery. The fundamental difference is that additional illusory disks or patches are reported in the HG, rather than a failure to report shape properties (features) that are present in the periphery. In this sense, the HC and EX are the opposite of the HG effect: They show a disappearance rather than an appearance of features.

## A Dissociation Between HC and EX

We devised a procedure that allows observers to directly report the extent of the visual field within which the lines or the disks are visible. We used this procedure to measure the strength of the illusions and to test the role of contrast polarity. As mentioned there is some evidence that high contrast between the grid and the features contributes to the EX (Levine & McAnany, 2008), but not to the HC (Bertamini et al., 2016). If this dissociation is confirmed within a study that directly compares the two illusions, it would suggest the existence of different mechanisms.

**Figure 2.**
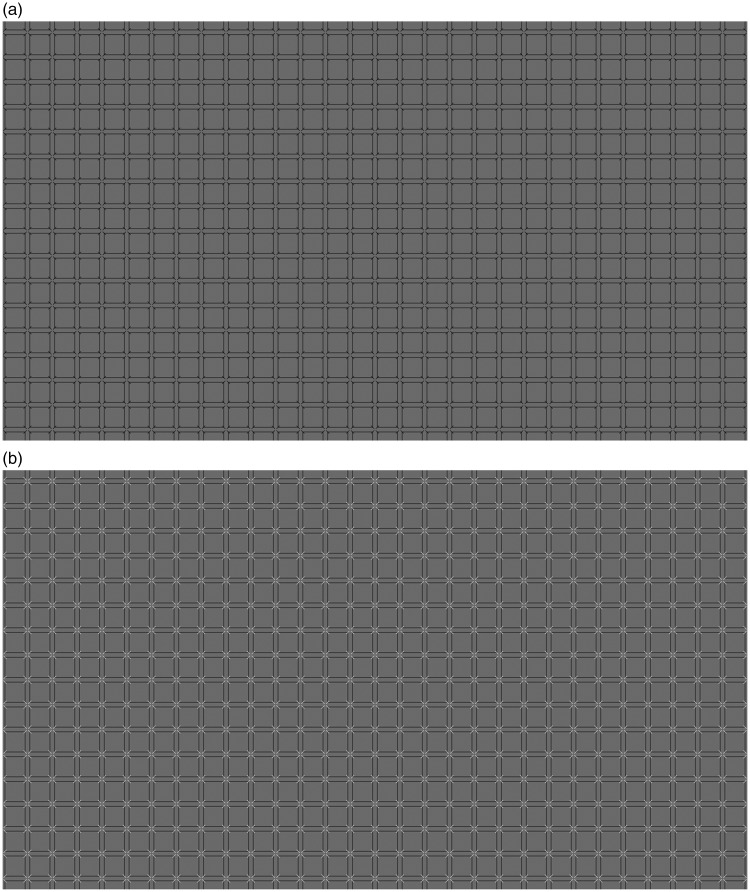
In this version of the Honeycomb illusion, squares replaced hexagons. In one version, the lines were black (a), and in another, the lines were white (b).

**Figure 3.**
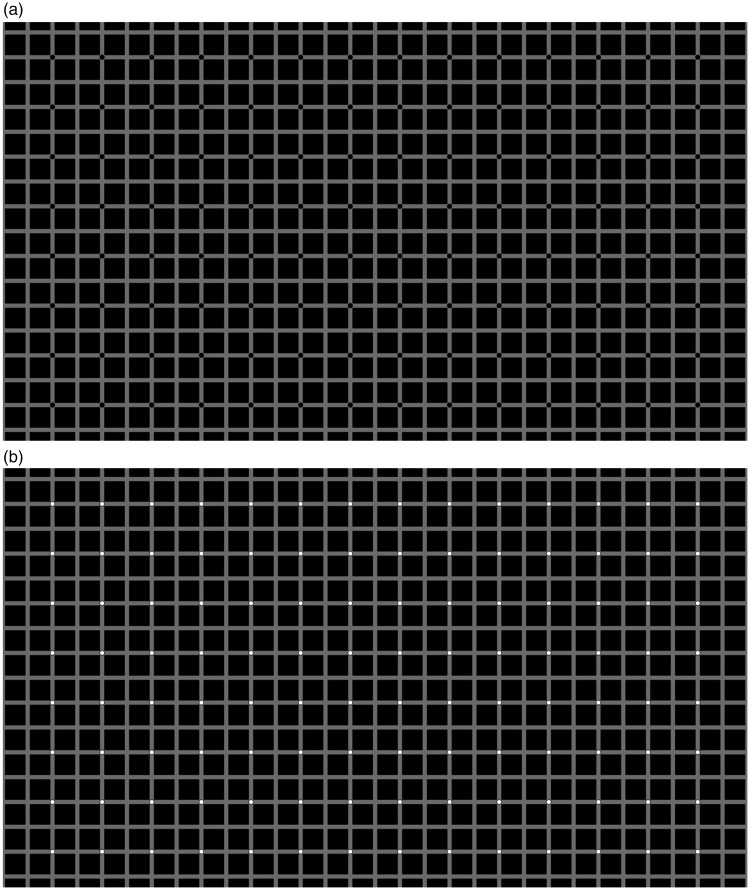
The Extinction illusion (a). In one version, the disks were black (a), and in the other, disks were white (b).

In terms of stimuli, we modified the original HC and EX illusions so as to make them more similar. In both cases, the underlying grid was made of squares. The size was the same and the only difference was that for the HC illusion the squares were outlines, with additional small lines at the corners, and for the EX illusion the squares were filled, and disks were placed at some of the intersections. Examples are shown in Figures 2 and 3.

During the study, there was a presentation of the background grid without the lines or the disks, a brief additional presentation of these features (lines or disks) and then an adjustment of a circular gauge allowing the observer to report the extent of the area in which the features were visible. We acknowledge that this is a type of self-report procedure and therefore subject to possible biases. We chose it so that observers can use the circle to provide a report of their phenomenal experience. Moreover, we interleaved several conditions, including control stimuli for which we expect no illusion, thus providing a baseline.

## Individual Differences in HC and EX

There has been great interest recently on the use of visual illusions to study individual differences and the existence of common factors (Grzeczkowski, Clarke, Francis, Mast, & Herzog, 2017). Susceptibility to illusions may vary in different clinical conditions, such as autism spectrum disorders (ASD) or dyslexia (Gori, Molteni, & Facoetti, 2016; Slaghuis, Twell, & Kingston, 1996), and there are other factors, including physiological (Peterzell & Kennedy, 2016) or cultural (de Fockert, Davidoff, Fagot, Parron, & Goldstein, 2007), which contribute to individual differences.

Illusions relating to awareness of stimulus properties in the periphery may be especially useful in relation to individual differences. They tell us something about visual awareness and show how available information, from memory or from central vision, is (or is not) used to construct a representation of the whole visual field experience.

For our study, we tested a sample of 56 observers. They were all undergraduate students, and therefore drawn from a restricted population. Most of them were females. We administered two standard questionnaires: the short version of the Oxford-Liverpool Inventory of Feelings and Experiences (short O-LIFE) and the Autism-Spectrum Quotient (AQ) questionnaire. The short O-LIFE (Mason, Linney, & Claridge, 2005) is intended for use in the general population to test for schizotypical traits and it includes three subscales of schizotypy; unusual symptoms, cognitive disorganisation and introvertive anhedonia. Some studies have found a decreased susceptibility to visual illusions in schizophrenia (for a review, see Notredame, Pins, Deneve, & Jardri, 2014), others (e.g., Grzeczkowski et al., 2018) found no difference in illusion strength between healthy controls and schizophrenic patients (see also Kaliuzhna et al., 2018). In the specific case of these illusions (in the visual periphery), it may be more relevant that individuals high in schizotypy have a response bias toward seeing something that is not there or that is not visible (Partos, Cropper, & Rawlings, 2016).

With respect to the AQ, there is a vast literature on ASD and visual perception (Baron-Cohen, Wheelwright, Skinner, Martin, & Clubley, 2001). A dissociation between psychosis and autism has been proposed by some authors (Crespi & Badcock, 2008). The AQ includes five subscales: social skills, attention switching, attention to detail, communication and imagination. It has been claimed that ASD individuals have weak global processing and a local over global advantage (Gori et al., 2016; Happé 1996; for reviews, see Simmons et al., 2009). We expect a negative correlation between AQ scores and the strength of the illusion. This prediction follows from the idea that attention to detail may reduce the phenomenal awareness of elements in the periphery. Reduced susceptibility to classic visual illusions has been reported in children with ASD (Happé, 1996), although Manning, Morgan, Allen, and Pellicano (2017) found that this was the case only when a method of adjustment was used. Note that our study does use a method of adjustment.

A more general consideration is that visual illusions are often reported as demonstrations or on the bases of reports from small samples. In the existing literature, this is true for both the HC and the EX illusions. Our study tested the robustness of these phenomena within a relatively large sample, although limited to undergraduate students, and we analysed, in exploratory fashion, the individual differences measured by two standard questionnaires (O-LIFE and AQ).

## Methods

### Observers

Fifty-six adults participated (54 females). All observers had normal or corrected-to-normal visual acuity. They were all undergraduate students at the University of Liverpool and were unaware of the purpose of the study. The study was approved by the local Ethics committee and written consent was obtained from all observers.

### Stimuli and Apparatus

The images were generated using Python and PsychoPy (Peirce, 2007) on a Macintosh computer and displayed on a Sony monitor (52.80 × 29.70 cm). The images filled the screen and had always a red cross in the centre. A chinrest was used to fix the distance from the screen at 57 cm.

**Figure 4.**
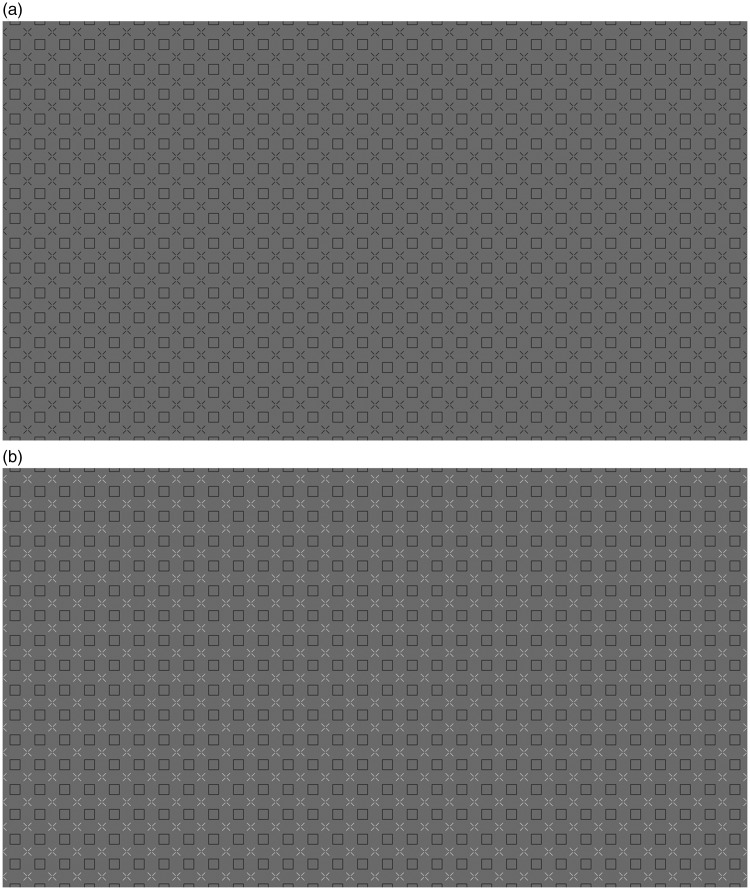
This is a control condition for the Honeycomb illusion in which the squares do not touch the lines. In one version, the lines were black (a), and in the other, lines were white (b).

**Figure 5.**
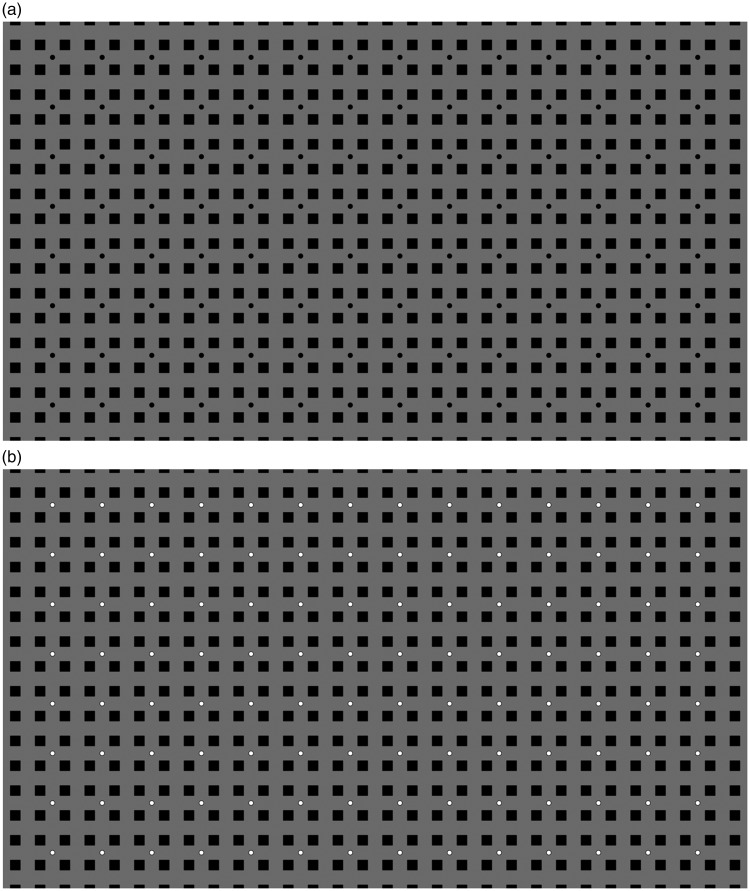
This is a control condition for the Extinction illusion in which the squares do not touch the disks. In one version, the disks were black (a), and in the other, disks were white (b).

The images for the HC illusion are shown in Figure 2, and the images for the EX illusion are shown in Figure 3. The background was always grey (luminance 44.5 cd/m^2^), although in the EX case much of the background was covered by black squares (luminance 2.9 cd/m^2^). The squares that formed the grid could be of two sizes: 0.43° or 0.86° of visual angle (25.8 and 51.6 arcmin). We refer to these as small and large square conditions. The features (lines or disks) were black or white in two different conditions. We refer to this as contrast polarity because it is a change in opposite direction relative to the grey background. Figures 2 and 3 show the large squares images. Figures 4 and 5 show the small squares images. The small squares stimuli were control conditions because with smaller squares the lines and the disks are not touching the squares. This is expected to lead to a reduced illusion.

The study included two illusions (HC and EX) two square sizes (large and small), two contrast polarity of the features (black and white) and three presentation durations (250, 500 and 750 ms). There were, therefore, 24 unique conditions. Each was presented 3 times for a total of 72 trials per observer.

### Procedure

**Figure 6.**
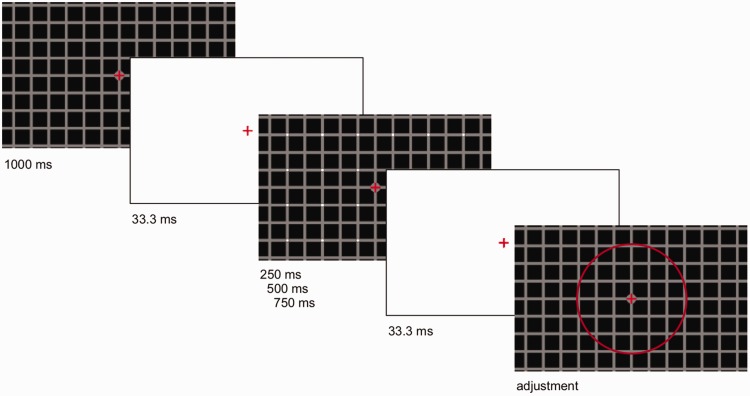
The procedure included a long presentation of the grid and a fixed period of presentation of the additional lines or disks. White screens were also included to minimise the role of luminance onset in the detection of features. The final image shows the red circle used for the adjustment. To make the disks visible in the figure, these images are cropped and show only a central region of the stimulus.

Each observer was tested individually in a dark and quite room. The texture made of squares was on the screen before the lines (HC) or disks (EX) were added and also at the time of the response. The sequence of events is illustrated in Figure 6. The grid was first presented for 1,000 ms. High luminance blank transients (white screen) were shown for 33.3 ms and the texture with the lines/disks for a variable amount of time based on condition. The luminance for the white screen was 230.0 cd/m^2^. There were three presentation durations: 250 ms, 500 ms and 750 ms.

In the response period, observers used a game controller to adjust a circle (the right and left buttons could make it increase or decrease in size). They could use a different button to report that they could see the lines (HC) or disks (EX) over the entirety of the image. There was no time pressure and after they were happy with the response they had to press another button on the controller to advance to the next trial. During the experiment, participants used a chinrest to keep them 57 cm away from the screen. Viewing was binocular.

### Data Preprocessing

To check for intrarater reliability, we computed two-way mixed-effects models—intraclass correlations of type (3,1) or ICC (3,1)—on the raw data set for each condition, as suggested in Shrout and Fleiss (1979) and in Koo and Li (2016).

For all conditions, the three adjustments of each observer were averaged. We then computed z-scores for each of the two size conditions. All data were within the ±3 standard deviations of the mean range.

## Results

### Intrarater Reliability

All intraclass correlations were significant (Bonferroni corrected), indicating that participants made consistent adjustments between all three trials of each condition (Table 1).

**Table 1.** All Intraclass Correlations Were Significant (Bonferroni Corrected) for Each Condition With 95% Confidence Intervals Excluding Zero, Indicating Good Intrarater Reliability Over Three Trials.

	EXBlack250S	EXBlack500S	EXBlack750S	EXWhite250S	EXWhite500S	EXWhite750S
ICC (3,1)	0.463	0.574	0.445	0.652	0.595	0.543
*F* value	3.589***	5.046***	3.407***	6.616***	5.406***	4.565***
95% CI	[0.303, 0.615]	[0.427, 0.704]	[0.283, 0.600]	[0.520, 0.764]	[0.451, 0.720]	[0.391,0.680]
	HCBlack250S	HCBlack500S	HCBlack750S	HCWhite250S	HCWhite500S	HCWhite750S
ICC (3,1)	0.462	0.558	0.703	0.467	0.664	0.501
*F* value	3.573***	4.790***	8.094***	3.630***	6.929***	4.010***
95% CI	[0.301, 0.614]	[0.409, 0.692]	[0.583, 0.801]	[0.307, 0.619]	[0.535, 0.773]	[0.344, 0.646]
	EXBlack250L	EXBlack500L	EXBlack750L	EXWhite250L	EXWhite500L	EXWhite750L
ICC (3,1)	0.369	0.408	0.407	0.410	0.433	0.384
*F* value	2.751***	3.071***	3.056***	3.082***	3.290***	2.868***
95% CI	[0.203, 0.534]	[0.244, 0.569]	[0.243, 0.567]	[0.246, 0.570]	[0.270, 0.590]	[0.219, 0.548]
	HCBlack250L	HCBlack500L	HCBlack750L	HCWhite250L	HCWhite500L	HCWhite750L
ICC (3,1)	0.552	0.394	0.330	0.459	0.548	0.543
*F* value	4.695***	2.950***	2.479***	3.550***	4.631***	4.569***
95% CI	[0.402, 0.687]	[0.229, 0.557]	[0.164, 0.500]	[0.299, 0.612]	[0.397, 0.684]	[0.392, 0.680]

*Note*. ‘250’, ‘500’ and ‘750’ indicate the presentation durations (in milliseconds); EX = Extinction illusion; HC = Honeycomb illusion; S = small square conditions; L = large square conditions; ICC = intraclass correlation; CI = confidence interval.

***p < .001.

### Small Square Conditions

Small square conditions were included as a control to demonstrate that the difficulty of seeing the lines or the disks was due to an interaction with the squares and not simply due to eccentricity. Since observers could also report that lines and disks were visible over the whole screen, data were inevitably truncated at screen size.

**Figure 7.**
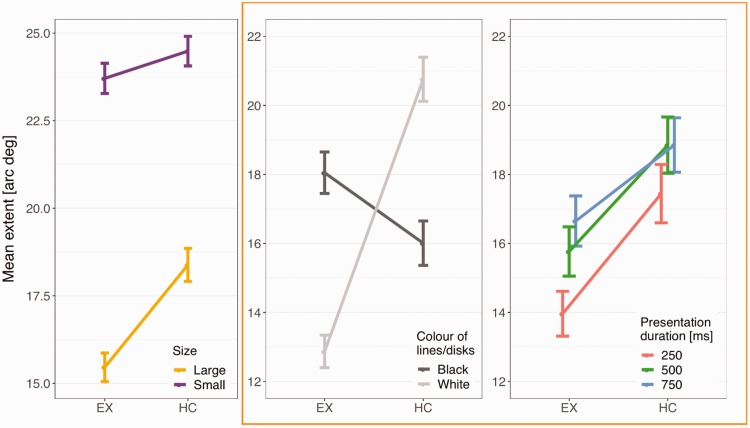
Mean extent of the region (in visual angle) in which lines and disks were visible (left panel) as a function of illusion for small versus large square conditions, (middle panel) as a function of illusion and contrast polarity, and (right panel) as a function of illusion and presentation duration. Only data from the large squares condition were included in the middle and right panels. Error bars show standard error of the mean.

Figure 7 (left panel) shows the mean extent of the region in which lines and disks are visible for the two illusions (HC and EX) separately for small and large square conditions. Strong ceiling effects were observed for the small square conditions. A paired *t* test (two-tailed) comparing the small square conditions to the large square conditions resulted in a significant difference, *t*(671) = 22.458, *p* < .001, suggesting that the illusion was reduced when the features (lines or disks) did not touch the squares. Only large square conditions were considered for further analyses.

### Large Square Conditions

The mean values from the large square conditions are plotted in the middle and right panels of Figure 7 (within an orange rectangle). As observed in the middle panel, there was a significant interaction between the illusion and contrast polarity. Importantly, this interaction might even be stronger than what we report, because the measured responses for the HC illusion with white lines were affected by a ceiling effect.

### Mixed-Effects Model

To account for random variations in baseline among participants, mixed-effects models were used (lmer R package; Bates, Mächler, Bolker, & Walker, 2014) in *RStudio* version 1.1.456 (RStudio Team, 2016). The dependent measure was the standardised scores. Illusion, contrast polarity (black or white lines/disks) and presentation duration were fixed effects—or predictors—and the nature of their relationships, that is, additive or interactive, was tested through log likelihood-ratio comparisons (χ^2^). We also tested the impact of the AQ and O-LIFE scores on the illusory effects. The marginal and conditional *r*^2^ effect sizes are reported as measures of the variance explained by the model with the random effect structure included (conditional *r*^2^) and excluded (marginal *r*^2^) from the calculation (Johnson, 2014; Nakagawa & Schielzeth, 2012). They both were computed from the MuMIn R package.

First, the interaction between illusion and contrast polarity was tested. As expected, a likelihood-ratio test revealed a significant difference between additive and interactive models, χ^2^(1) = 132.470, *p* < .001, therefore suggesting a significant interaction between illusion and contrast polarity (Figure 7, middle panel).

Second, the model did not significantly improve, χ^2^(6) = 7.573, *p* = .271, when presentation duration interacted with illusion and contrast polarity (illusion × contrast polarity). However, the presentation duration predictor significantly improved the model, χ^2^(2) = 18.303, *p* < .001. Hence, there seems to be an effect of presentation duration on the extent of the region in which lines and disks are visible in both illusions (Figure 7, right panel), with a slight increase in the extent of the region visible when presentation duration increases. Third, the total AQ and O-LIFE scores did not significantly improve the model—AQ: χ^2^(1) = 0.063, *p* = .801; O-LIFE: χ^2^(1) = 0.036, *p* = .850.

The best model therefore included an interaction between illusion and contrast polarity, together with presentation duration. This model accounted for 13.6% of the variance in the data without the random effects, but 58.2% when they were included ($r_{m}^{\text{2}}$ = .136; $r_{c}^{\text{2}}$ = .582). The estimates for the individual levels of fixed effects are shown in Table 2.

**Table 2.** Estimates From The Mixed-Effects Model With Illusion, Contrast Polarity (Black Lines/Disks or White Lines/Disks) and Presentation Duration as Predictors (Interaction Between Illusion and Contrast Polarity).

Fixed Effects	*Β* Estimate	*Β* Standard Error	*t* Value
Intercept	−0.011	0.108	−0.101
Honeycomb illusion	−0.249	0.071	−3.536
White lines/disks	−0.632	0.071	−8.968
500 ms presentation duration	0.196	0.061	3.210
750 ms presentation duration	0.250	0.061	4.096
Honeycomb Illusion × White Lines/Disks	1.212	0.100	12.157

Only data from large square conditions were included in the model.

### Correlations

Pairwise correlations were computed between the large square conditions and AQ and O-LIFE scores. Results are reported both with Bonferroni correction and without correction (Table 3). The HC and EX illusions were significantly correlated across contrast polarity and presentation durations, suggesting a similar pattern of individual differences for the two illusions. However, there were no significant correlations between the large square conditions and AQ or O-LIFE scores (except for a weak negative correlation between HCWhite500 and O-LIFE score which did however not survive Bonferroni correction: *r* = −.266, *p* = .048).

**Table 3.** Correlations Between Large Square Conditions and AQ and O-LIFE Scores Expressed as Correlation Coefficients (Pearson’s *r*).

	*r*	1	2	3	4	5	6	7	8	9	10	11	12	13	14
1	EXBlack250		0.674	0.683	0.717	0.516	0.519	0.745	0.542	0.629	0.493	0.492	0.505	0.047	0.127
2	EXBlack500			0.734	0.604	0.541	0.511	0.623	0.409	0.446	0.469	0.477	0.427	0.146	0.040
3	EXBlack750				0.433	0.375	0.388	0.469	0.348	0.391	0.271	0.386	0.369	0.016	0.050
4	EXWhite250					0.684	0.655	0.725	0.547	0.612	0.559	0.471	0.429	0.129	0.140
5	EXWhite500						0.752	0.569	0.426	0.453	0.473	0.472	0.428	0.080	0.114
6	EXWhite750							0.533	0.485	0.521	0.493	0.442	0.469	−0.048	0.094
7	HCBlack250								0.712	0.768	0.537	0.443	0.340	0.003	0.001
8	HCBlack500									0.818	0.522	0.489	0.364	−0.104	−0.129
9	HCBlack750										0.466	0.464	0.341	−0.066	0.010
10	HCWhite250											0.816	0.703	−0.171	−0.226
11	HCWhite500												0.801	−0.223	−0.266
12	HCWhite750													−0.021	−0.046
13	AQ														0.678
14	O-LIFE														

*Note*. Light grey indicates significance without any correction, while dark grey indicates significance after Bonferroni correction was applied. Abbreviations are further explained in Table 1.

## Discussion

In the HC and the EX illusions, textures that are uniform appear non-uniform. We have introduced a procedure that allows observers to directly report the extent of the visual field over which they were able to see some features (lines or disks). The texture was presented first without lines or disks; then these features were briefly introduced and then removed. After that observers adjusted the radius of a circle to report the percept. This relies on introspection. There are both advantages and weaknesses to this methodology, sometimes referred to as experimental phenomenology (Kubovy, 1999; Kubovy & Gepshtein, 2002).

In our study, it was important to include a baseline condition. We did that by reducing the size of the square elements so that there was no connection between the squares and the lines or disks. We confirmed that this increased the visibility of the features. Therefore, the lines and disks themselves are clearly visible in the periphery and spatial resolution per se is not the reason why the lines are invisible in the HC illusion. Hence, a specific interaction between the squares and the lines must render the lines invisible. The same arguments hold true for the disks in the EX illusion.

In crowding, identification of a target deteriorates in the presence of flankers. Contrast polarity plays a crucial role. Crowding is reduced when flanker and target have opposite contrast polarity compared to when they have the same polarity (Herzog, Sayim, Chicherov, & Manassi, 2015; Kooi, Toet, Tripathy, & Levi, 1994). This role of contrast polarity is similar to what happens with the EX illusion. However, crowding does not render elements invisible. Usually, elements appear as distorted, jumbled or superimposed on each other (Pelli, Palomares, & Majaj, 2004). Although our procedure does not provide direct evidence, we are not aware of any evidence of perceived distortions in the HC or EX illusions. The lines are simply invisible outside a small central region. In addition, crowding is not due to simple flanker target interactions of the type we may have between squares and lines (e.g., Herzog & Manassi, 2015). It may be that the lines are invisible because of some sort of contrast reduction or normalisation, which the larger squares induce. Still, the question arises why the brain does not fill in the lines as it does in some filling-in phenomena or in the healing grid illusion. In the case of filling-in of the blind spot, for example, lines are perceived as complete and not reduced in length (Tripathy, Levi, Ogmen, & Harden, 1995). Patches that differ in colour or motion with respect to the background also are filled in with the background information after fading (Ramachandran & Gregory, 1991), and the lack of sensitivity to blue of the human foveola also leads to filling-in (Magnussen, Spillmann, Stürzel, & Werner, 2001). Moreover, a recent study has found that a foveal percept, even when not veridical (central vision filling-in in scotopic vision), was relied upon more than peripheral information (Gloriani & Schütz, 2019). In other words, there are several phenomena where the brain uses available information, in particular at fixation, to fill-in another region of the visual field. In particular, when comparing stimuli near and far from fixation, there is a bias to rely more on the information near fixation (Gloriani & Schütz, 2019). In the HC and EX illusions, we see the reverse, information at fixation is not used for a uniform percept that extends beyond fixation. The mechanism that makes disks and lines invisible in the periphery is stronger than any bias in favour of central vision as well as any prior in favour of uniformity.

In the introduction, we briefly discussed the literature on ensemble perception. In the periphery, similar items may be coded only in terms of summary statistics (Haberman & Whitney, 2012; Whitney & Yamanashi Leib, 2018). In general terms, one could describe the HC and the EX illusions as special cases of ensemble perception. They are special cases because they suggest that the representation of what is in the periphery follows its own rigid rules. It is the rigidity of these processes that creates different representations of the same texture at fixation and away from fixation.

In our study, we have compared directly the HC and the EX illusions. The conditions were similar in that the size of the squares was the same, the task was the same and the trials were interleaved. In line with the original description of these effects, the lines were on each square (HC illusion, Figure 2), while the disks were not present at every intersection (EX illusion, Figure 3). We manipulated presentation time and confirmed that this factor was not critical, although the strength of the illusion decreased with presentation time. We were particularly interested in the role of contrast polarity. As predicted, contrast polarity (white disks among black squares) produced a stronger EX illusion (smaller region of visibility), but contrast polarity (white lines over black squares) produced a weaker HC illusion (larger region of visibility). This type of dissociation suggests that the mechanisms at play are either different in the two cases or, more likely, they dependent on contrast polarity in a complex way that is affected by the spatial relationship between targets (lines and disks) and texture (squares).

Overall in our sample (*N* = 56), the effect was clearer (smaller visibility region) for the EX than HC illusion. This difference is not important as the illusions rely on qualitatively different stimuli. More interesting is the fact that contrast polarity strongly modulated the effect (a cross-over interaction). Responses were consistent over the trials (intrarater reliability was good) and the two illusions correlated with each other. These correlations suggest that individual differences are stable between the two illusions and across changes in presentation duration. Overall, the evidence is that both illusions exist in the majority of individuals, that their effects are larger than in the control conditions when the squares were small, and that they are affected by contrast polarity. The fact that contrast polarity had opposite effects requires further work and provides strong constraints for models. The way information from the periphery relies on summary statistics may play a role here, but we are not aware of a model that can explain the disappearance of some objects and not others, and the way the two illusions depend on contrast polarity.

Finally, we considered individual differences. The sample was entirely constituted by undergraduate students, mostly females. We were interested in whether susceptibility to the illusions (as measured by the size of the visibility region) increases with O-LIFE scores and decreases with AQ scores. The link with schizotypy may be mediated by a response bias toward reporting something that is not visible (Partos et al., 2016), and the link with ASD spectrum may be mediated by a focus on the local details (Jolliffe & Baron-Cohen, 1997). These hypotheses were not confirmed. Instead, in line with what was found by Russell-Smith, Maybery, and Bayliss (2011), O-LIFE and AQ scores were positively correlated with each other and unrelated to the strength of the illusion. As noted in the introduction, some recent reports have failed to find clear correlations between the strength of illusions and clinical conditions, for instance, schizophrenia and schizotypy (Grzeczkowski et al., 2017, 2018).

We conclude by returning to a few general considerations about what makes these illusions so informative and revealing. Both the HC and the EX illusions are counterexamples to a version of the Grand illusion hypothesis. The illusion of seeing a rich and detailed visual field cannot be simply the result of accumulated information over multiple fixations. It is also a counterexample to the idea of an assumption of uniformity for textures, which could explain some of the phenomenal richness of our visual experience. We need to develop more sophisticated models of how visual information determines what observers perceive as extended textures. Surprisingly, even when the textures or patterns are uniform, they may appear as non-uniform.
